# Variable selection in generalized random coefficient autoregressive models

**DOI:** 10.1186/s13660-018-1680-4

**Published:** 2018-04-12

**Authors:** Zhiwen Zhao, Yangping Liu, Cuixin Peng

**Affiliations:** 1 College of Mathematics, Jilin Normal University, Siping, P.R. China; 2 Public Foreign Languages Department, Jilin Normal University, Siping, P.R. China

**Keywords:** 62M10, 91B62, Empirical likelihood, Akaike information criterion, Bayesian information criterion, Generalized random coefficient autoregressive model, Variable selection

## Abstract

In this paper, we consider the variable selection problem of the generalized random coefficient autoregressive model (GRCA). Instead of parametric likelihood, we use non-parametric empirical likelihood in the information theoretic approach. We propose an empirical likelihood-based Akaike information criterion (AIC) and a Bayesian information criterion (BIC).

## Introduction

Consider the following *p*-order generalized random coefficient autoregressive model:

1 $$ Y_{t}=\Phi_{t}^{\tau }Y(t-1)+ \varepsilon_{t}, $$

where *τ* denotes the transpose of a matrix or vector, $\Phi_{t}=( \Phi_{t1},\ldots ,\Phi_{tp})^{\tau }$ is a random coefficient vector, $Y(t-1)=(Y_{t-1}, \ldots ,Y_{t-p})^{\tau }$, and $\{\binom{\Phi_{t}}{\varepsilon_{t}}, t=0,\pm 1, \pm 2, \ldots \}$ is a sequence of i.i.d. random vectors with $E(\Phi_{t})=\phi =(\phi_{1},\ldots ,\phi_{p})$, $E(\varepsilon _{t})=0$, and $\operatorname{Var}\binom{\Phi_{t}}{\varepsilon_{t}} = \bigl( {\scriptsize\begin{matrix}{} V_{\phi }& \sigma_{\Phi \varepsilon }\cr \sigma_{\Phi \varepsilon }^{\tau }& \sigma_{\varepsilon }^{2} \end{matrix}}\bigr)$.

As a generalization of the usual autoregressive model, the random coefficient autoregressive (RCAR) model (*cf.* [1, 2]), the Markovian bilinear model and its generalization, and the random coefficient exponential autoregressive model (*cf.* [3–5]), model (1) was first introduced by Hwang and Basawa [6]. GRCA has become one of the important models in the nonlinear time series context. In recent years, GRCA has been studied by many authors. For instance, Hwang and Basawa [7] established the local asymptotic normality of a class of generalized random coefficient autoregressive processes. Carrasco and Chen [8] provided the tractable sufficient conditions that simultaneously imply strict stationarity, finiteness of higher-order moments, and *β*-mixing with geometric decay rates. Zhao and Wang [9] constructed confidence regions for the parameters of model (1) by using an empirical likelihood method. Furthermore, Zhao et al. [10] also considered the problem of testing the constancy of the coefficients in the stationary one-order generalized random coefficient autoregressive model. In this paper, we consider the variable selection problem of the GRCA based on the empirical likelihood method.

Many model selection procedures have been proposed in the statistical literature, including the adjusted $R^{2}$ (see Theil [11]), the AIC (see Akaike [12]), BIC (see Schwarz [13]), Mallow’S $C_{p}$ (see Mallows [14]). Other criteria in the literature include Hannan and Quinn’s criterion [15], Geweke and Meese’s criterion [16], Cavanaugh’s Kullback information criterion [17], and the deviance information criterion of Spiegelhalter et al. [18]. Also, Tsay [19], Hurvich and Tsai [20] and Pötscher [21] have studied model selection methods in time series models. Recently, the model selection problem has been extended to moment selection as in Andrews [22], Andrews and Lu [23] and Hong et al. [24]. These model selection methods are concerned with parsimony, as was stressed in Zellner et al. [25], as well as accuracy or power in choosing models.

In this paper, we develop an information theoretic approach to variable selection problem of GRCA. Specifically, instead of parametric likelihood, we use non-parametric empirical likelihood (see Owen [26, 27]) in the information theoretic approach. We propose an empirical likelihood-based Akaike information criterion (EAIC) and a Bayesian information criterion (EBIC).

The paper proceeds as follows. The next section is concerned with the methodology and the main results. Section 3 is devoted to the proofs of the main results.

Throughout the paper, we use the symbols “$\stackrel{d}{\longrightarrow}$” and “$\stackrel{p}{\longrightarrow }$” to denote convergence in distribution and convergence in probability, respectively. We abbreviate “almost surely” and “independent identical distributed” to “a.s.” and “i.i.d.”, respectively. $o_{p}(1)$ means a term which converges to zero in probability. $O_{p}(1)$ means a term which is bounded in probability. Furthermore, the Kronecker product of the matrices *A* and *B* is denoted by $A \otimes B$, and $\Vert M \Vert $ denotes the $L_{2}$ norm for vector or matrix *M*.

## Methods and main results

In this section, we will first propose the empirical likelihood-based information criteria for choice of a GRCA, then we investigate the asymptotic properties of the new variable selection method.

### Empirical likelihood-based information criteria

Hwang and Basawa [6] derived the conditional least-squares estimator *ϕ̂* of *ϕ*, which is given by

$$\begin{aligned}& \hat{\phi } = \Biggl(\sum_{t=1}^{n}Y(t-1)Y^{\tau }(t-1) \Biggr)^{-1} \Biggl(\sum_{t=1} ^{n}Y_{t}Y(t-1) \Biggr). \end{aligned}$$

By using the estimating equation of the conditional least-squares estimator, we can obtain the following score function:

$$ \sum_{t=1}^{n} \bigl(Y_{t}Y(t-1)-Y(t-1)Y^{\tau }(t-1) \phi \bigr)=\sum_{t=1}^{n}G_{t}( \phi ), $$

where $G_{t}(\phi )=Y_{t}Y(t-1)-Y(t-1)Y^{\tau }(t-1)\phi $. Following Owen [26], the empirical likelihood statistic for *ϕ* is defined as

$$ \tilde{l}(\phi )=-2\max_{\sum_{t=1}^{n}p_{t}G_{t}(\phi )=0}\sum _{t=1} ^{n}\log (np_{t}), $$

where $p_{1},\ldots , p_{n}$ are all sets of nonnegative numbers summing to 1. By using the Lagrange multiplier method, let

$$ G=\sum_{t=1}^{n}\log (np_{t})-n \lambda^{\tau }\sum_{t=1} ^{n}p_{t}G_{t}( \phi )+\gamma \Biggl(\sum_{t=1}^{n}p_{t}-1 \Biggr). $$

After simple algebraic calculation, we have

$$ \frac{\partial G}{\partial p_{t}}=\frac{1}{p_{t}}-n\lambda^{\tau }G _{t}(\phi )+\gamma , \quad t=1,\ldots , n. $$

Note that $\sum_{t=1}^{n}p_{t}=1$ and $\sum_{t=1}^{n}p _{t}G_{t}(\phi )=0 $. So we have $\gamma =-n$ and $p_{t}=\frac{1}{n(1+ \lambda^{\tau }G_{t}(\phi ))}$, which implies that

2 $$ \tilde{l}(\phi )=2\sum_{t=1}^{n} \log \bigl(1+\lambda^{\tau }G_{t}( \phi ) \bigr), $$

where *λ* is the solution of the equation

3 $$ \frac{1}{n}\sum_{t=1}^{n} \frac{G_{t}(\phi )}{1+\lambda^{\tau }G _{t}(\phi )}=0. $$

The definition of $\tilde{l}(\phi )$ relies on finding a positive ${p_{t}}'s$ such that $\sum_{t=1}^{n}p_{t}G_{t}(\phi )=0$ for each *ϕ*. The solution exists if and only if the convex hull of the $G_{t}(\phi )$, $t=1, 2, \ldots , n$ contains zero as an inner point. When the model is correct, the solution exists with probability tending to 1 as the sample size $n\rightarrow \infty $ for *ϕ* in a neighborhood of $\phi_{0}$. However, for finite *n* and at some *ϕ* value, the equation often does not have a solution in $p_{t}$. To avoid this problem, we introduce the adjusted empirical likelihood.

Further let $\bar{G}_{n}=n^{-1}\sum_{t=1}^{n}p_{t}G_{t}(\phi )$ and define ${G}_{n+1}=-a_{n}\bar{G}_{n}$ for some positive constant $a_{n}$. We adjust the profile empirical log-likelihood ratio function to

4 $$\begin{aligned} l(\phi )&=-2\max_{\sum_{t=1}^{n+1}p_{t}G_{t}(\phi )=0} \sum_{t=1}^{n+1} \log \bigl({(n+1)}p_{t} \bigr) \\ &=2\sum_{t=1}^{n+1}\log \bigl\{ 1+\tilde{ \lambda }^{\tau }G_{t}( \phi ) \bigr\} \end{aligned}$$

with $\tilde{\lambda }=\tilde{\lambda }(\phi )$ being the solution of

5 $$ \frac{1}{n+1}\sum_{t=1}^{n+1} \frac{G_{t}(\phi )}{1+\lambda^{ \tau }G_{t}(\phi )}=0. $$

Since 0 always lies on the line connecting $\bar{G}_{n}$ and ${G}_{n+1}$, the adjusted empirical log-likelihood ratio function is well defined after adding a pseudo-value ${G}_{n+1}$ to the data set. The adjustment is particularly useful so that a numerical program does not crash simply because some undesirable *ϕ* is assessed.

A full GRCA assumes that $y_{t}$ relates to $\Phi_{t}^{\tau }Y(t-1)$ with $E(\Phi_{t})=\phi $ being unknown parameter of size *p*. Let *s* be a subset of $\{1, 2, \ldots , p\}$, and $Y^{[s]}(t-1)$ and $\phi^{[s]}$ be subvectors of $Y(t-1)$ and *ϕ* containing entries in positions specified by *s*. Consider the *p*th-order GRCA specified by $E(G_{t}(\phi ))=0$ and a submodel specified by $E(G^{[s]}_{t}( \phi^{[s]}))=0$, where $G^{[s]}_{t}(\phi^{[s]})=Y_{t}Y^{[s]}(t-1)-Y ^{[s]}(t-1)(Y^{[s]}(t-1))^{\tau }\phi^{[s]}$. For a given *s*, let $G^{[s]}_{t}=Y_{t}Y^{[s]}(t-1)-Y^{[s]}(t-1)(Y^{[s]}(t-1))^{\tau } \phi^{[s]}$, $\bar{G}_{n}^{[s]}=n^{-1}\sum_{t=1}^{n}G^{[s]} _{t}$ and $G^{[s]}_{n+1}=-a_{n}\bar{G}^{[s]}_{n}$ for some positive constant $a_{n}$. The adjusted empirical log-likelihood ratio becomes

6 $$\begin{aligned} l \bigl(\phi^{[s]} \bigr)&=-2\max_{\sum_{t=1}^{n+1}p_{t}G^{[s]}_{t}=0} \sum _{t=1}^{n+1}\log \bigl({(n+1)}p_{t} \bigr) \\ &=2\sum_{t=1}^{n+1}\log \bigl\{ 1+\tilde{ \lambda }^{\tau }G^{[s]} _{t} \bigr\} \end{aligned}$$

with $\tilde{\lambda }=\tilde{\lambda }(\phi )$ being the solution of

7 $$ \frac{1}{n+1}\sum_{t=1}^{n+1} \frac{G^{[s]}_{t}}{1+\lambda^{ \tau }G^{[s]}_{t}}=0. $$

We define the adjusted profile empirical log-likelihood ratio as

8 $$ l(s)=\inf \bigl\{ l \bigl(\phi^{[s]} \bigr): \phi^{[s]} \bigr\} . $$

The empirical likelihood versions of AIC and BIC are then defined as

9 $$\begin{aligned}& \operatorname{EAIC}=l(s)+2k, \end{aligned}$$

10 $$\begin{aligned}& \operatorname{EBIC}=l(s)+k\log (n), \end{aligned}$$

where *k* is the cardinality of *s*.

After $l(s)$ is evaluated for all s, we select the model with the minimum EAIC or EBIC value.

### Asymptotic properties

It is well known that under some mild conditions the parametric BIC is consistent for variable selection while the parametric AIC is not. Similarly, we can prove that, when p is constant, EBIC is consistent but EAIC is not.

For purposes of illustration, in what follows, we rewrite the model in the following matrix form (see Hwang and Basawa [6]): let $U_{t}=(\varepsilon_{t},0,0,\ldots ,0)^{\tau }$ are $p\times 1$ vectors, $\tilde{\Phi }_{tj}={\Phi }_{tj}-\phi_{j}$, $j=1,\ldots ,p$,

$$\begin{aligned} B=\left ( \textstyle\begin{array}{@{}c@{\quad}c@{\quad}c@{\quad}c@{\quad}c@{}} \phi_{1} & \phi_{2} & \cdots & \phi_{p-1} & \phi_{p} \\ 1 & 0 & \cdots & 0 & 0 \\ 0& 1 & \cdots & 0 & 0 \\ \cdots & \cdots & \cdots & \cdots & \cdots \\ 0 & 0 & \cdots & 1 & 0 \end{array}\displaystyle \right ) _{p\times p},\qquad C_{t}= \left ( \textstyle\begin{array}{@{}c@{\quad}c@{\quad}c@{\quad}c@{}} \tilde{\Phi }_{t1} & \tilde{\Phi }_{t2} & \cdots & \tilde{\Phi }_{tp} \\ 0 & 0 & \cdots & 0 \\ 0& 0 & \cdots & 0 \\ \cdots & \cdots & \cdots & \cdots \\ 0 & 0 & \cdots & 0 \end{array}\displaystyle \right ) _{p\times p} . \end{aligned}$$

Then model (1) can be written as

11 $$ Y(t)=(B+C_{t})Y(t-1)+ U_{t}. $$

In order to obtain our theorems, we need the following regularity conditions:

$\mathbf{(A_{1})}$
All the eigenvalues of the matrix $E(C_{t}\otimes C _{t})+(B\otimes B)$ are less than unity in modulus.
$\mathbf{(A_{2})}$
$EY_{t}^{6}<\infty $.

#### Remark 1

As for the condition $\mathbf{(A_{1})}$ and the sufficient condition for $E\vert y_{t} \vert ^{2m}<\infty$ ($m=1, 2, \ldots$), we refer to Hwang and Basawa [6].

#### Theorem 2.1

*Let* $A=E(G_{t}(\phi_{0})G^{\tau }_{t}(\phi_{0}))$ *and* $B=E((\partial G_{t}(\phi )/\partial \phi )|_{\phi =\phi_{0}})$. *If* $\mathbf{(A_{1})}$ *and* $\mathbf{(A_{2})}$ *hold*, *then there exists a sequence of adjusted empirical likelihood estimates* *ϕ̃* *of* *ϕ* *such that*

12 $$ \sqrt{n}(\tilde{\phi }-\phi )\rightarrow N \bigl(0, \bigl(B^{\tau }A^{-1}B \bigr)^{-1} \bigr) $$

*and*

13 $$ \sqrt{n}(\tilde{\lambda }-\lambda )\rightarrow N(0,U), $$

*where* $U=A^{-1}-A^{-1}B(B^{\tau }A^{-1}B)^{-1}B^{\tau }A^{-1}$.

Note that when a submodel *s* is a true model, it implies $\phi_{0} ^{[\bar{s}]}=0$. That is, components of $\phi_{0}$ not in *s* are zero. Therefore, $Y_{t}$ only relates to the variables in positions specified by *s*. The following theorem shows that when $\phi_{0}^{[\bar{s}]}=0$ is true, then adjusted empirical log-likelihood ratio statistic has a chi-squared limiting distribution with *k* fewer degrees of freedom.

#### Theorem 2.2

*Assume that* $\mathbf{(A_{1})}$ *and* $\mathbf{(A_{2})}$ *hold and* $\phi_{0} ^{[\bar{s}]}=0$ *for a submodel* *s* *of size k*. *Then when* $a_{n}=o _{p}(n^{\frac{1}{2}})$, *we have* $l(s)\rightarrow \chi^{2}_{p-k}$ *in distribution as* $n\rightarrow \infty $.

When the null hypothesis of $\phi_{0}^{[\bar{s}]}=0$ is not true, the likelihood ratio go to ∞ as $n\rightarrow \infty $. We state the following theorem in terms of the adjusted empirical likelihood which also applies to the usual empirical likelihood.

#### Theorem 2.3

*Assume that* $\mathbf{(A_{1})}$ *and* $\mathbf{(A_{2})}$ *hold and* $a_{n}=o_{p}(n ^{\frac{1}{2}})$. *Then for any* $\phi \neq \phi_{0}$ *such that* $E(G_{t}(\phi ))\neq 0$, $l(s)\rightarrow \infty $ *in probability as* $n\rightarrow \infty $.

The following theorem indicates that, when *p* is constant, EBIC is consistent but EAIC is not.

#### Theorem 2.4

*Assume that* $\mathbf{(A_{1})}$ *and* $\mathbf{(A_{2})}$ *hold and if there exists a subset* $s_{0}$ *of* $1, 2, \ldots , p$ *such that*, *for any other subset* *s*, $E(G^{[s]}_{t}(\phi^{[s]}))=0$ *for some* *ϕ* *if and only if* *s* *contains* $s_{0}$. *Then*, *EBIC is consistent and EAIC is not consistent*.

## Proofs of the main results

In order to prove Theorem 2.1, we first present several lemmas.

### Lemma 3.1

*Assume that* $\mathbf{(A_{1})}$ *and* $\mathbf{(A_{2})}$ *hold*. *Then* *A* *is positive definite and* *B* *has rank p*.

### Proof

After simple algebra calculation, we have, for any nonzero vector $c=(c_{1}, \ldots , c_{p})\in R^{P}$,

$$\begin{aligned}& c^{\tau }Ac =E \bigl(c^{\tau }G_{t}(\phi )G^{\tau }_{t}(\phi )c \bigr)=E \bigl( \bigl(c^{\tau }Y(t-1) \bigr)^{2}\operatorname{Var} \bigl(Y_{t}| Y(t-1) \bigr) \bigr). \end{aligned}$$

Note that the conditional distribution of $Y_{t}$, given $Y(t-1)$, is not a degenerate distribution, which implies that $\operatorname{Var}(Y_{t}| Y(t-1))>0 $ a.s. It follows that $(c^{\tau }Y(t-1))^{2}\operatorname{Var}(Y_{t}| Y(t-1)) \geq 0 $ a.s. Therefore, $c^{\tau }Ac=0$ if and only if $c^{\tau }Y(t-1)=0$ a.s. Without loss of generality, suppose that the first component $c_{1}$ of *c* is 1, so $Y_{t-1}=-c_{2}Y_{t-2}-\cdots -c_{p}Y_{t-p}$, which is contradictory with the fact that the conditional distribution of $Y_{t-1}$, given $(Y_{t-2}, \ldots , Y _{t-p})$, is not degenerate. Hence $c^{\tau }Ac>0$. That is, *A* is positive definite.

Similarly, we can also prove that *B* has rank *p*. The proof of Lemma 3.1 is thus complete. □

### Lemma 3.2

*Assume that* $\mathbf{(A_{1})}$ *and* $\mathbf{(A_{2})}$ *hold*. *Then when* $a_{n}=o(n^{\frac{1}{2}})$, *we have*

14 $$\begin{aligned}& \sup_{\phi }\Biggl\Vert \frac{1}{n+1}\sum_{t=1}^{n+1}G_{t}(\phi)G^{\tau }_{t}(\phi ) \Biggr\Vert =O(1)\quad (a.s.), \end{aligned}$$

*uniformly about* $\phi \in \{\phi | \Vert \phi -\phi_{0} \Vert \leq n^{-\frac{1}{3}}\}$.

### Proof

Note that

15 $$\begin{aligned} &\sup_{\phi } \Biggl\Vert \frac{1}{n+1}\sum_{t=1}^{n+1}G_{t}( \phi)G^{\tau }_{t}(\phi ) \Biggr\Vert \\ &\quad \leq \sup_{\phi }\Biggl\Vert \frac{1}{n+1}\sum _{t=1}^{n}G_{t}( \phi)G^{\tau }_{t}(\phi ) \Biggr\Vert +\sup _{\phi } \frac{1}{n+1}a_{n} ^{2} \Biggl\Vert \frac{1}{n} \sum_{t=1}^{n}G_{t}( \phi ) \Biggr\Vert ^{2} \\ &\quad \leq \sup_{\phi }\Biggl\Vert \frac{1}{n+1}\sum _{t=1}^{n}G_{t}( \phi)G^{\tau }_{t}(\phi )-\frac{1}{n+1}\sum _{t=1}^{n}G_{t}(\phi_{0})G^{\tau }_{t}( \phi_{0}) \Biggr\Vert \\ &\quad \quad {}+\Biggl\Vert \frac{1}{n+1}\sum_{t=1}^{n}G_{t}( \phi_{0})G^{\tau }_{t}(\phi_{0}) \Biggr\Vert +\sup_{\phi }\frac{1}{n+1}a_{n}^{2} \Biggl\Vert \frac{1}{n}\sum_{t=1}^{n}G_{t}( \phi ) \Biggr\Vert ^{2} \\ &\quad \triangleq L_{n1}+L_{n2}+L_{n3}. \end{aligned}$$

First, note that

$$\begin{aligned} L_{n1} &=\sup_{\phi }\Biggl\Vert \frac{1}{n+1}\sum_{t=1}^{n} \bigl(Y(t-1)Y^{\tau }(t-1) \bigl(Y^{\tau }(t-1) (\phi - \phi_{0}) \bigr) \bigr) \Biggr\Vert \\ &\leq \frac{1}{n+1}\sum_{t=1}^{n} \bigl\Vert Y(t-1) \bigr\Vert ^{3} \sup_{\phi } \Vert \phi -\phi_{0} \Vert . \end{aligned}$$

By the ergodic theorem, we have

16 $$\begin{aligned}& \frac{1}{n+1}\sum_{t=1}^{n} \bigl\Vert Y(t-1) \bigr\Vert ^{3}=O(1) \quad (a.s.). \end{aligned}$$

Further, note that

17 $$\begin{aligned}& \sup_{\phi }\Vert \phi -\phi_{0} \Vert =O(1). \end{aligned}$$

This, together with (16), proves that

18 $$\begin{aligned}& L_{n1} =O \bigl(n^{-\frac{1}{3}} \bigr) \quad (a.s.). \end{aligned}$$

Again by the ergodic theorem, we can prove that

19 $$\begin{aligned}& L_{n2} =O(1)\quad (a.s.). \end{aligned}$$

Finally, we prove that

20 $$\begin{aligned}& L_{n3} =O \bigl(n^{-\frac{1}{3}} \bigr) \quad (a.s.). \end{aligned}$$

Note that

$$\begin{aligned}& \sup_{\phi } \Biggl\Vert \frac{1}{n}\sum _{t=1}^{n}G_{t}(\phi ) \Biggr\Vert \leq \sup_{\phi }\Biggl\Vert \frac{1}{n}\sum_{t=1}^{n} \bigl(G_{t}(\phi )-G^{\tau }_{t}(\phi_{0}) \bigr) \Biggr\Vert + \Biggl\Vert \frac{1}{n}\sum _{t=1}^{n}G_{t}(\phi_{0}) \Biggr\Vert . \end{aligned}$$

Similar to the proof of (18), we can show that

21 $$\begin{aligned}& \sup_{\phi }\Biggl\Vert \frac{1}{n}\sum _{t=1}^{n} \bigl(G_{t}( \phi)-G^{\tau }_{t}(\phi_{0}) \bigr) \Biggr\Vert =O \bigl(n^{-\frac{1}{3}} \bigr)\quad (a.s.). \end{aligned}$$

In what follows, we consider $\Vert \frac{1}{n}\sum_{t=1}^{n}G_{t}(\phi_{0}) \Vert $.

Denote the *i*th component of $G_{t}(\phi_{0})$ by $G_{ti}(\phi_{0})$. Then $\{G_{ti}(\phi_{0}), 1\leq i\leq p\}$ is a stationary ergodic martingale difference sequence with $E(G_{ti}(\phi_{0}))=0$ and $E((G_{ti}(\phi_{0}))^{2})<\infty $. By the law of the iterated logarithm of martingale difference sequence, we have, for $1\leq i \leq p$,

$$ \frac{1}{n}\sum_{t=1}^{n}G^{\tau }_{ti}( \phi_{0})= O \bigl(n^{- \frac{1}{2}} \bigl(\log_{2}^{n} \bigr)^{\frac{1}{2}} \bigr) \quad (a.s.). $$

It follows that

22 $$\begin{aligned}& \frac{1}{n}\sum_{t=1}^{n}G^{\tau }_{t}( \phi_{0})= O \bigl(n^{- \frac{1}{2}} \bigl(\log_{2}^{n} \bigr)^{\frac{1}{2}} \bigr) \quad (a.s.). \end{aligned}$$

Then, by (21) and (22), we have

23 $$\begin{aligned}& \sup_{\phi } \Biggl\Vert \frac{1}{n}\sum _{t=1}^{n}G_{t}(\phi ) \Biggr\Vert =O \bigl(n ^{-\frac{1}{3}} \bigr)\quad (a.s.). \end{aligned}$$

Therefore

24 $$\begin{aligned} L_{n3} &=O \bigl(n^{-1} \bigr)o \bigl(n^{\frac{1}{2}} \bigr)o \bigl(n^{\frac{1}{2}} \bigr)O \bigl(n^{- \frac{1}{3}} \bigr)O \bigl(n^{-\frac{1}{3}} \bigr)\quad (a.s.) \\ &=o \bigl(n^{-\frac{2}{3}} \bigr)\quad (a.s.). \end{aligned}$$

This, together with (18) and (19), proves Lemma 3.2. □

### Lemma 3.3

*Assume that* $\mathbf{(A_{1})}$ *and* $\mathbf{(A_{2})}$ *hold*. *Then when* $a_{n}=o(n^{\frac{1}{2}})$, *we have*

25 $$ \max_{1\leq t\leq {n+1}}\sup_{\phi } \bigl\Vert G_{t}(\phi ) \bigr\Vert =o \bigl(n ^{\frac{1}{3}} \bigr) \quad (a.s.), $$

*uniformly about* $\phi \in \{\phi | \Vert \phi -\phi_{0} \Vert \leq n^{-\frac{1}{3}}\}$.

### Proof

Note that

$$\begin{aligned} \max_{1\leq t\leq {n+1}}\sup_{\phi } \bigl\Vert G_{t}(\phi ) \bigr\Vert &\leq\max_{1\leq t\leq {n}}\sup_{\phi } \bigl\Vert G_{t}( \phi ) \bigr\Vert + \sup_{\phi }\Biggl\Vert a_{n} \frac{1}{n}\sum_{t=1}^{n}G_{t}( \phi ) \Biggr\Vert \\ &\triangleq K_{n1}+K_{n2}. \end{aligned}$$

From (23), together with $a_{n}=o(n^{\frac{1}{2}})$, it follows immediately that

26 $$\begin{aligned}& K_{n2} =o \bigl(n^{\frac{1}{3}} \bigr)\quad (a.s.). \end{aligned}$$

The next step in the proof is to show that

27 $$\begin{aligned}& K_{n1} =o \bigl(n^{\frac{1}{3}} \bigr) \quad (a.s.). \end{aligned}$$

By the Fubini theorem, we have, for any positive integer *k*,

$$\begin{aligned} \infty&>E \Bigl(\sup_{\phi } \bigl\Vert G_{t}(\phi ) \bigr\Vert \Bigr)^{3} \\ &=\int_{0}^{\infty }P \Bigl( \Bigl(\sup _{\phi } \bigl\Vert G_{t}(\phi ) \bigr\Vert \Bigr)^{3}>s \Bigr)\,ds \\ &=\sum_{n=1}^{\infty } \int_{(n-1)k^{3}}^{nk^{3}} P \Bigl( \Bigl(\sup _{\phi } \bigl\Vert G_{t}(\phi ) \bigr\Vert \Bigr)^{3}>s \Bigr)\,ds \\ &\geq\sum_{n=1}^{\infty } \int_{(n-1)k^{3}}^{nk^{3}} P \Bigl( \Bigl(\sup _{\phi } \bigl\Vert G_{t}(\phi ) \bigr\Vert \Bigr)^{3}>nk^{3} \Bigr)\,ds \\ &=\sum_{n=1}^{\infty }P \Bigl( \Bigl(\sup_{\phi } \bigl\Vert G_{t}(\phi ) \bigr\Vert \Bigr)^{3}>nk ^{3} \Bigr)k^{3}\,ds. \end{aligned}$$

Thus, using the ergodic theorem,

28 $$ \sum_{n=1}^{\infty }P \Bigl(\sup _{\phi } \bigl\Vert G_{n}(\phi ) \bigr\Vert >n ^{\frac{1}{3}}k \Bigr) < \infty . $$

By the Borel–Cantelli lemma, we know that

29 $$ P \Bigl(\sup_{\phi } \bigl\Vert G_{n}( \phi ) \bigr\Vert >n^{\frac{1}{3}}k \mbox{ i.o.}\Bigr)=0, $$

so that

30 $$ \sup_{\phi } \bigl\Vert G_{n}(\phi ) \bigr\Vert \leq n^{\frac{1}{3}}k \quad (a.s.). $$

Take $k=\frac{1}{m}$, then there exists $Q_{m}$ with $P(Q_{m})=0$, such that, for any $\omega \in Q_{m}^{c}$,

31 $$ \frac{\sup_{\phi }\Vert G_{n}(\phi ) \Vert }{n^{\frac{1}{3}}}\leq \frac{1}{m}. $$

Further, let $Q=\bigcup_{m=1}^{\infty }Q_{m}$. Then

32 $$ \lim_{n\rightarrow \infty }\frac{\sup_{\phi }\Vert G_{n}(\phi ) \Vert }{n^{\frac{1}{3}}}=0, $$

which, together with the fact that $P(Q)=0$, implies that

33 $$ \max_{1\leq t\leq {n}}\sup_{\phi } \bigl\Vert G_{t}(\phi ) \bigr\Vert =o \bigl(n ^{\frac{1}{3}} \bigr) \quad (a.s.). $$

The proof is complete. □

### Lemma 3.4

*Assume that* $\mathbf{(A_{1})}$ *and* $\mathbf{(A_{2})}$ *hold*. *Then when* $a_{n}=o(n^{\frac{1}{2}})$, *we have*

34 $$ \sup_{\phi } \bigl\Vert \lambda (\phi ) \bigr\Vert =O \bigl(n^{-\frac{1}{3}} \bigr)\quad (a.s.), $$

*uniformly about* $\phi \in \{\phi | \Vert \phi -\phi_{0} \Vert \leq n^{-\frac{1}{3}}\}$.

### Proof

Write $\Vert \lambda (\phi ) \Vert =\rho (\phi )\theta (\phi )$, where $\rho (\phi )>0$ and $\Vert \theta (\phi ) \Vert =1$. Further let

35 $$ Q_{1,n+1}(\phi ,\lambda )=\frac{1}{n+1}\sum _{t=1}^{n+1}\frac{G _{t}(\phi )}{1+\lambda^{\tau }(\phi ) G_{t}(\phi )}. $$

Then

$$\begin{aligned} 0&= \bigl\Vert Q_{1,n+1}(\phi , \lambda ) \bigr\Vert \\ &\geq \Biggl\vert \frac{1}{n+1}\sum _{t=1}^{n+1}\frac{\theta^{\tau }(\phi )G_{t}(\phi )}{1+\lambda^{\tau }(\phi ) G_{t}(\phi )} \Biggr\vert \\ &\geq \Biggl\vert \frac{1}{n+1}\rho (\phi )\sum _{t=1}^{n+1}\frac{\theta^{\tau }(\phi )G_{t}(\phi )G^{\tau }_{t}(\phi )\theta (\phi )}{1+\rho (\phi )\theta^{\tau }(\phi ) G_{t}(\phi )} \Biggr\vert - \Biggl\vert \frac{1}{n+1}\sum_{t=1}^{n+1} \theta^{\tau }(\phi )G_{t}(\phi ) \Biggr\vert \\ &\geq \frac{\rho (\phi )\theta^{\tau }(\phi )(\frac{1}{n+1}\sum_{t=1} ^{n+1}G_{t}(\phi )G^{\tau }_{t}(\phi ))\theta (\phi )}{\max_{1\leq t\leq n}\{1+\rho (\phi )\theta^{\tau }(\phi ) G_{t}( \phi )\}}-\Biggl\vert \frac{1}{n+1}\sum _{t=1}^{n+1}\theta^{\tau }(\phi )G_{t}( \phi ) \Biggr\vert , \end{aligned}$$

which implies that

36 $$\begin{aligned} \frac{\rho (\phi )\theta^{\tau }(\phi )(\frac{1}{n+1}\sum_{t=1} ^{n+1}G_{t}(\phi )G^{\tau }_{t}(\phi ))\theta (\phi )}{\max_{1\leq t\leq n}\{1+\rho (\phi )\theta^{\tau }(\phi ) G_{t}( \phi )\}} &\leq\Biggl\vert \frac{1}{n+1}\sum_{t=1}^{n+1} \theta^{\tau }(\phi)G_{t}(\phi ) \Biggr\vert \\ &\leq \Biggl\Vert \frac{1}{n+1}\sum_{t=1}^{n+1}G_{t}( \phi ) \Biggr\Vert . \end{aligned}$$

Further, by the ergodic theorem, we have

37 $$ \Biggl\Vert \frac{1}{n+1}\sum_{t=1}^{n+1}G_{t}( \phi_{0})G^{\tau }_{t}(\phi_{0})-A \Biggr\Vert =o(1)\quad (a.s.), $$

where $A=E(G_{t}(\phi_{0})G^{\tau }_{t}(\phi_{0}))$.

Since

$$\begin{aligned} 0&\leq\sup_{\phi }\Biggl\Vert \frac{1}{n+1}\sum_{t=1}^{n+1}G_{t}( \phi )G^{\tau }_{t}(\phi )-A \Biggr\Vert \\ &\leq \sup_{\phi }\Biggl\Vert \frac{1}{n+1}\sum_{t=1}^{n+1}G_{t}( \phi)G^{\tau }_{t}(\phi )-\frac{1}{n+1}\sum _{t=1}^{n+1}G_{t}(\phi_{0})G^{\tau }_{t}( \phi_{0}) \Biggr\Vert \\ &\quad {}+\Biggl\Vert \frac{1}{n+1}\sum _{t=1}^{n+1}G_{t}(\phi_{0})G^{\tau }_{t}( \phi_{0})-A \Biggr\Vert , \end{aligned}$$

we have from (18) and (37)

38 $$ \frac{1}{n+1}\sum_{t=1}^{n+1}G_{t}( \phi )G^{\tau }_{t}(\phi )=A+o(1)\quad (a.s.), $$

which implies that

39 $$ \theta^{\tau }(\phi ) \Biggl(\frac{1}{n+1}\sum _{t=1}^{n+1}G_{t}( \phi )G^{\tau }_{t}( \phi ) \Biggr)\theta (\phi )\geq \sigma_{\min }+o(1)\quad (a.s.), $$

where $\sigma_{\min }$ is the smallest eigenvalue and the largest eigenvalue of *A*. This, together with Lemma 3.1 and (36), proves that

$$\begin{aligned}& \sup_{\phi }\Biggl\Vert \frac{1}{n+1}\sum_{t=1}^{n+1}G_{t}( \phi ) \Biggr\Vert \\& \quad \geq \sup_{\phi }\rho (\phi ) \Biggl( \sigma_{\min }+o(1)- \Bigl(\max_{1\leq t\leq {n+1}}\sup _{\phi } \bigl\Vert G_{t}(\phi ) \bigr\Vert \Bigr) \Biggl( \sup_{\phi } \Biggl\Vert \frac{1}{n+1}\sum _{t=1}^{n+1}G_{t}(\phi ) \Biggr\Vert \Biggr) \Biggr). \end{aligned}$$

Combined with (23) and Lemma 3.3, this establish (34) and completes the proof. □

### Lemma 3.5

*Assume that* $\mathbf{(A_{1})}$ *and* $\mathbf{(A_{2})}$ *hold*, *and* $a_{n}=o _{(}n^{\frac{1}{2}})$. *Then*, *as* $n\rightarrow \infty $, *with probability* 1, $l(\phi )$ *attains its minimum value at some point* *ϕ̃* *in the interior of the ball* $\Vert \phi -\phi_{0} \Vert \leq n^{-\frac{1}{3}}$ *and* *ϕ̃* *and* $\tilde{\lambda }=\lambda (\tilde{\phi })$ *satisfy* $Q_{1,n+1}(\tilde{\phi },\tilde{\lambda })=0$ *and* $Q_{2,n+1}( \tilde{\phi },\tilde{\lambda })=0$, *where* $Q_{1,n+1}(\phi ,\lambda )$ *is defined in* (35) *and*

40 $$ Q_{2n}(\phi ,\lambda ) =\frac{1}{n+1}\sum _{t=1}^{n+1}\frac{1}{1+ \lambda^{\tau }G_{t}(\phi )} \biggl( \frac{\partial G_{t}(\phi )}{\partial \phi } \biggr)^{\tau }\lambda . $$

The proof is similar to the proof of Lemma 1 of Qin and Lawless [28], so we omit the details.

### Proof of Theorem 2.1

In what follows, we omit $(\phi ,\lambda )$ in the notation if a function is evaluated at $(\phi_{0}, 0)$. Expanding $Q_{1,n+1}(\tilde{\phi }, \tilde{\lambda })$, $Q_{2,n+1}(\tilde{\phi },\tilde{\lambda })$ at $(\phi_{0}, 0)$ leads to

41 $$ 0=Q_{1,n+1}(\tilde{\phi },\tilde{\lambda })=Q_{1,n+1}+ \biggl\{ \frac{\partial Q_{1,n+1}}{\partial \phi } \biggr\} ( \tilde{\phi }- \phi_{0})+ \biggl\{ \frac{\partial Q_{1,n+1}}{\partial \lambda } \biggr\} \tilde{\lambda }+o_{p}(\delta_{n}) $$

and

42 $$ 0=Q_{2,n+1}(\tilde{\phi },\tilde{\lambda }) =Q_{2,n+1}+ \biggl\{ \frac{\partial Q_{2,n+1}}{\partial \phi } \biggr\} ( \tilde{\phi }- \phi_{0})+ \biggl\{ \frac{\partial Q_{2,n+1}}{\partial \lambda } \biggr\} \tilde{\lambda }+o_{p}(\delta_{n}), $$

where $\delta_{n}=\Vert \tilde{\phi }-\phi_{0} \Vert ^{2}+\Vert \tilde{\lambda } \Vert ^{2}=O_{p}(n^{-\frac{2}{3}})$.

Note that

43 $$\begin{aligned}& \frac{\partial Q_{1,n+1}}{\partial \phi } = \frac{1}{n+1}\sum _{t=1}^{n+1}\frac{\partial G_{t}}{\partial \phi }=B+o_{p}(1), \end{aligned}$$

44 $$\begin{aligned}& \frac{\partial Q_{1,n+1}}{\partial \lambda } = -\frac{1}{n+1}\sum_{t=1}^{n+1}G_{t}G^{\tau }_{t}=-A+o_{p}(1), \end{aligned}$$

45 $$\begin{aligned}& \frac{\partial Q_{2,n+1}}{\partial \phi } = 0, \end{aligned}$$

and

46 $$ \frac{\partial Q_{1,n+1}}{\partial \lambda } =\frac{1}{n+1}\sum _{t=1}^{n+1}\frac{\partial G_{t}}{\partial \phi }=B^{\tau }+o _{p}(1). $$

These, combined with (41) and (42), give

47 $$ \tilde{\lambda } =- \bigl\{ A^{-1}-A^{-1}B \bigl(B^{\tau }A^{-1}B \bigr)^{-1}B^{\tau }A ^{-1} \bigr\} Q_{1,n+1}+o_{p} \bigl(n^{-\frac{1}{2}} \bigr) $$

and

48 $$ \tilde{\phi }-\phi_{0} = \bigl(B^{\tau }A^{-1}B \bigr)^{-1}B^{\tau }A^{-1}Q_{1,n+1}+o _{p} \bigl(n^{-\frac{1}{2}} \bigr). $$

Further, applying the central limit theorem to $Q_{1,n+1}$ and using Slustzky’s theorem, we can prove Theorem 2.1. □

### Proof of Theorem 2.2

Let *λ̃* be the Lagrange multiplier corresponding to $\tilde{\phi }^{[s]}$, the maximum point of $l(\phi^{[s]})$. With this notation, we may write

49 $$ l(s) =2\sum_{t=1}^{n+1}\log \bigl\{ 1+\tilde{\lambda }^{\tau }G^{[s]} _{t} \bigl( \tilde{\phi }^{[s]} \bigr) \bigr\} . $$

Note that

50 $$ \tilde{\lambda }^{\tau }G^{[s]}_{t} \bigl(\tilde{\phi }^{[s]} \bigr)= \tilde{\lambda }^{\tau }G^{[s]}_{t}+ \tilde{\lambda }^{\tau } \biggl\{ \frac{ \partial G^{[s]}_{t}}{\partial \phi^{[s]} } \biggr\} ^{\tau } \bigl(\tilde{\phi } ^{[s]}-\phi_{0}^{[s]} \bigr)+o_{p}(1). $$

This, together with (49), yields

$$\begin{aligned} \l (s) &=2\tilde{\lambda }^{\tau }\sum_{t=1}^{n+1}G^{[s]}_{t}+2 \tilde{\lambda }^{\tau } \Biggl\{ \sum_{t=1}^{n+1}\frac{\partial G^{[s]}_{t}}{\partial \phi^{[s]} } \Biggr\} \bigl(\tilde{\phi }^{[s]}- \phi_{0}^{[s]} \bigr) - \tilde{\lambda }^{\tau }\sum_{t=1}^{n+1}G^{[s]}_{t} \bigl(G^{[s]} _{t} \bigr)^{\tau }+o_{p}(1) \tilde{\lambda } \\ &=n^{-1}Q_{1,n+1}^{\tau } \bigl\{ A^{-1}-A^{-1}B \bigl(B^{\tau }A^{-1}B \bigr)^{-1}B ^{\tau }A^{-1} \bigr\} Q_{1,n+1}+o_{p}(1). \end{aligned}$$

Further note that $Q_{1,n+1}$ is asymptotic normal with covariance matrix A and $\{A^{-1}-A^{-1}B(B^{\tau }A^{-1}B)^{-1}B^{\tau }A^{-1} \}A\{A^{-1}-A^{-1}B(B^{\tau }A^{-1}B)^{-1}B^{\tau }A^{-1}\}=\{A^{-1}-A^{-1}B(B^{\tau }A^{-1}B)^{-1}B^{\tau }A^{-1}\}$. Therefore, we have $\l (s)\rightarrow \chi^{2}(p-k)$ in distribution as $n\rightarrow \infty $. The proof is complete. □

### Proof of Theorem 2.3

Since $E(G_{t}(\phi ))\neq 0$, it follows that there exists $\delta >0$, such that

51 $$ \frac{1}{n}\sum_{t=1}^{n}G_{t}^{\tau }(\phi )\frac{1}{n} \sum_{t=1}^{n}G_{t}(\phi )-\delta^{2}=o_{p}(1). $$

Furthermore, note that $E(G_{t}^{\tau }(\phi ))^{2}<\infty $. Thus, by a similar method to the proof of (27), we can prove that

52 $$ \max_{1\leq t\leq n+1} \bigl\Vert G_{t}^{\tau }( \phi ) \bigr\Vert =o_{p} \bigl(n^{ \frac{1}{2}} \bigr). $$

Let $\check{\lambda }=n^{-\frac{2}{3}}(\frac{1}{n}\sum_{t=1} ^{n}G_{t}(\phi ))\log n$. Then

53 $$ \max_{1\leq t\leq n+1} \bigl\vert \check{\lambda }^{\tau }G_{t}(\phi ) \bigr\vert =o _{p}(1). $$

Thus, with probability going to 1, $1+\check{\lambda }^{\tau }G_{t}( \phi )>0$ for $i=1, \ldots , n+1$. Using the duality of the maximization problem and (51)–(53), we have

$$\begin{aligned} l(\phi ) &=\sup_{\lambda } \Biggl( 2\sum_{t=1}^{n+1}\log \bigl\{ 1+ \lambda^{\tau }G_{t}( \phi ) \bigr\} \Biggr)\geq 2\sum_{t=1}^{n+1}\log \bigl\{ 1+\check{ \lambda }^{\tau }G_{t}( \phi ) \bigr\} \\ &=2\sum_{t=1}^{n}\log \bigl\{ 1+\check{ \lambda }^{\tau }G_{t}( \phi ) \bigr\} +o_{p}(1)=2n^{\frac{1}{3}}\delta^{2}\log (n)+o_{p}(1), \end{aligned}$$

which implies that $l(s)\rightarrow \infty $ in probability as $n\rightarrow \infty $. The proof is complete. □

### Proof of Theorem 2.4.

First, we consider EAIC. Consider the situation when $s_{0}$ is empty. Let $s=\{1\}$ which contains a single covariant. Based on expansion in the proof of Theorem 2.2, we can prove that $l(s_{0})-l(s)\rightarrow \chi^{2}_{1}$, which implies that $\lim_{n\rightarrow \infty }P(l(s_{0})-l(s)>2)>0$. Therefore, EAIC is not consistent.

Next, we consider EBIC. Suppose *s* is a model which does not contain $s_{0}$. Then, $E(G^{[s]}_{t}(\phi^{[s]}))\neq 0$ for any $\phi^{[s]}$. Therefore, we have $l(s)\geq 2n^{\frac{1}{3}}\delta^{2}\log (n)+o _{P}(1)$. This order implies that

$$ P \bigl(\operatorname{EBIC}(s)< \operatorname{EBIC}(s_{0}) \bigr)\leq P \bigl(l(s)-l(s_{0})+p \log n \bigr)\rightarrow 0. $$

That is, EBIC will not select any model *s* that does not contain $s_{0}$.

Furthermore, if *s* contains $s_{0}$, and $k>0$ additional insignificant variables, by Theorem 2.2, we have

$$ l(s_{0})-l(s)\rightarrow \chi^{2}_{k}, $$

which implies that

$$ P \bigl(\operatorname{EBIC}(s)< \operatorname{EBIC}(s_{0}) \bigr)= P \bigl(l(s)-l(s_{0})>k \log n \bigr)\rightarrow 0, $$

as $n\rightarrow \infty $. Thus, the model *s* will not be selected by EBIC as $n\rightarrow \infty $. Because p is finite, there are only finite number of *s*competing against $s_{0}$, and each of them has $o(1)$ probability being selection. So EBIC is consistent. The proof is complete. □

## Conclusions

It should be pointed out that variable selection has always been an important problem for our statistician. Many variable selection methods have been proposed in the statistical literature. But for the variable selection method of GRCA, so far it has not been provided by statistician. In this paper, instead of parametric likelihood, we further propose an Akaike information criterion (EAIC) and a Bayesian information criterion (EBIC) for the variable selection problem of GRCA based on the empirical likelihood method. Moreover, we also prove that under some mild conditions the parametric EBIC is consistent, while the parametric EAIC is not when p is constant.
